# Altered phosphatidylcholines expression in sputum for diagnosis of non-small cell lung cancer

**DOI:** 10.18632/oncotarget.11283

**Published:** 2016-08-13

**Authors:** Jianyong Zhang, Jianjun Xu, Haiyan Lu, Jianhua Ding, Dongliang Yu, Penghui Li, Jianwen Xiong, Xingxing Liu, Huanwen Chen, Yiping Wei

**Affiliations:** ^1^ Department of Cardiothoracic Surgery, the Second Affiliated Hospital of Nanchang University, Nanchang, Jiangxi Province 330006, P. R. China; ^2^ Jiangxi Key Laboratory for Mass Spectrometry and Instrumentation, East China Institute of Technology, Nanchang, Jiangxi Province 330013, P. R. China

**Keywords:** NSCLC, sputum, phosphatidylcholines, ND-EESI-MS

## Abstract

Non–small cell lung cancer (NSCLC) is a leading cause of cancer mortality worldwide, and early diagnosis needs to be improved. We examined whether neutral desorption extractive electrospray ionization mass spectrometry (ND-EESI-MS) could be used to detect sputum lipids expression changes to enable earlier diagnosis. Overall, 167 NSCLC patients and 140 controls were enrolled. The main peaks in the sputum of patients with NSCLC patients differed from controls (83.3% of total variability), and the signals were not associated with pathological type, TNM stage or smoking history. The relative abundance of peaks at m/z734, m/ z756, m/z772, m/z782, m/z798 and m/z803 reliably distinguished NSCLC sputum from control. Collision-induced dissociation confirmed that m/z734, m/z756, and m/z772 represented [DPPC + H]^+^, [DPPC + Na]^+^, and [DPPC + K]^+^, respectively, and m/z782, m/z798, and m/z803 represented sphingomyelin, phosphatidylglycerol, and phosphatidylglycerolphosphate, respectively. The relative abundance of DPPC was clearly lower in NSCLC sputum than in control, and the relative abundances of phosphatidylglycerol and phosphatidylglycerolphosphate were higher in NSCLC sputum than in control. The detection of changes in sputum lipids with ND-EESI-MS may be a noninvasive, radiation-free, relatively inexpensive, repeatable, and efficient method for diagnosis of NSCLC.

## INTRODUCTION

Lung cancer is a leading cause of cancer death in both men and women world wide [1]. There are two types of lung cancer: small cell lung cancer (SCLC) and non–small cell lung cancer (NSCLC). NSCLC, which accounts for 75%–85% of all lung cancers, predominantly comprises adenocarcinomas, squamous cell carcinomas, and large cell lung carcinomas [2]. Currently, the 5-year relative survival rate for NSCLC is 10%–18%. These low rates are partly because more than 65% of cases are diagnosed at a late stage, when the 5-year survival rate is only about 5%. Therefore, there is an urgent need to improve the rate of early diagnosis of these tumors [3].

In many pulmonary diseases (e.g., tuberculosis, chronic obstructive pulmonary disease [COPD], cystic fibrosis, lung cancer, pneumonia and other bacterial pulmonary diseases), sputum examination is a valuable tool for disease diagnosis. The sputum has been the target for the discovery of noninvasive biomarkers to diagnose NSCLC because it contains airway epithelial cells, and molecular alterations identified in sputum are most likely to reflect tumor-associated changes [4]. However, the low sensitivity and reproducibility, and the necessity for complex pretreatment, has limited the application of this method in the diagnosis of NSCLC [4, 5].

Analysis of sputum by mass spectrometry (MS) may be one method to predict and assess NSCLC, as MS can provide rich molecular information on and in the sputum, which can be correlated with molecular biomarkers [4]. MS analysis, with its many advantages—including speed of analysis, high sensitivity, low limit of detection (LOD), and lack of requirement for analyte-specific reagents—is considered a powerful method for analyzing complex mixtures. In our earlier study, using tissue spray ionization mass spectrometry (TIS–MS), we have reported that dipalmitoyl PC [DPPC + Na]^+^ was higher in normal tissue than in cancerous tissue [6].

In this study, we used a novel ambient MS strategy termed neutral desorption extractive electrospray ionization mass spectrometry (ND-EESI-MS) that has been developed from extractive electrospray ionization mass spectrometry (EESI) and has been shown to be capable of detecting chemicals directly in viscous samples, including cosmetic, toothpaste, and honey [7–9]. With this method it is possible to analyze sputum directly.

## RESULTS

### ND-EESI-MS analysis of the sputum

**Figure 1:**
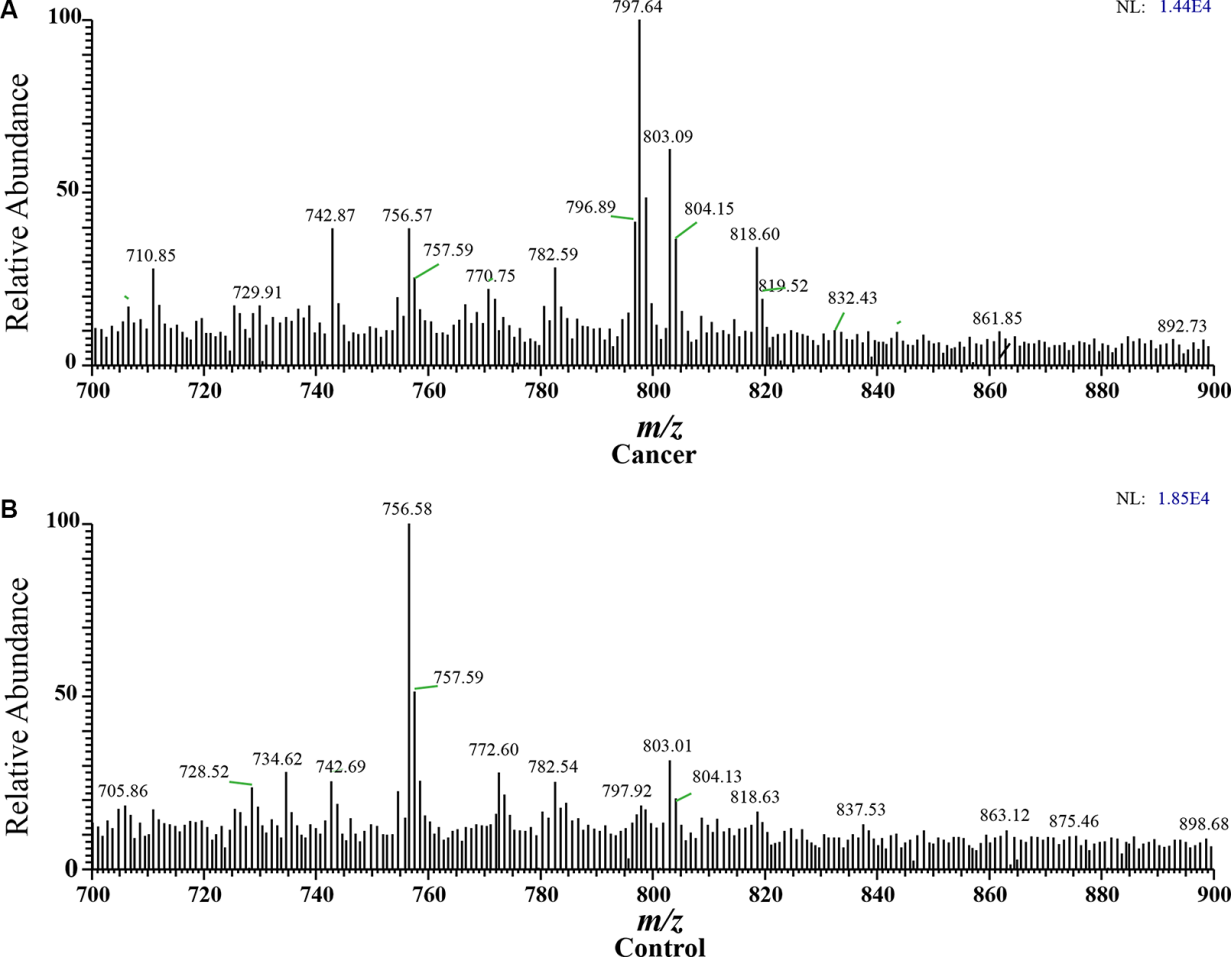
Spectra obtained from human sputum (**A**) Cancer: NSCLC sputum in positive ion mode, with main peaks at m/z710.85, m/z742.87, m/z756.57, m/z782.59 m/z797.64, m/z803.09 and m/z818.60, and so on. (**B**) Control: Control sputum in positive ion mode, with main peaks at m/z m/z 728.52, m/z 734.62, m/z 756.58, m/z 772.60, m/z782.54, m/z803.01, and so on.)

In the positive ion mode, the dominant species in the m/z range 700–900 are the phospholipids, and the main peaks are identified as protonated and sodium adducts of phosphatidylcholine. A systematic difference was clearly observed for ND-EESI-MS fingerprints collected from NSCLC sputum and control sputum. When a gentle N_2_ gas flow was driven into the desorption liquid to form a N_2_/methanol/acetic acid gas flow as neutral desorption airflow. The analytes of sputum were extracted when the neutral desorption airflow was driven into sputum. The ionizing solvent was delivered by a pump with a flow rate of 5 μL/ min, ionized by a high voltage of +3.5 kV, and atomized by a strong N_2_ gas flow. The interior analytes of the sputum were charged in the ionization region. The rich signals were caught when the charged analytes reached the mass spectrometer. In positive ion mode, in NSCLC sputum, the main peaks were at m/z710.85, m/z742.87, m/z756.57, m/ z782.59, m/z797.64, m/z803.09, and m/z818.60, and so on, while the main peaks were at m/z 728.52, m/z 734.62, m/z 756.58, m/z 772.60, m/z782.54, m/z803.01, and so on in control sputum. The relative abundances of m/z710.85, m/z742.87, m/z797.64, m/z803.09, and m/z818.60 were higher in NSCLC sputum, while the relative abundances of m/z 728.52, m/z 734.62, m/z 756.58, and m/z 772.60 were greater in control sputum (Figure 1). These signals in each sample were not associated with any clinical parameter, such as gender, age, tumor size and location in the lung, pathological type, TNM stage, or smoking history (Supplementary Figure S1–S5).

### Principal component analysis

**Figure 2:**
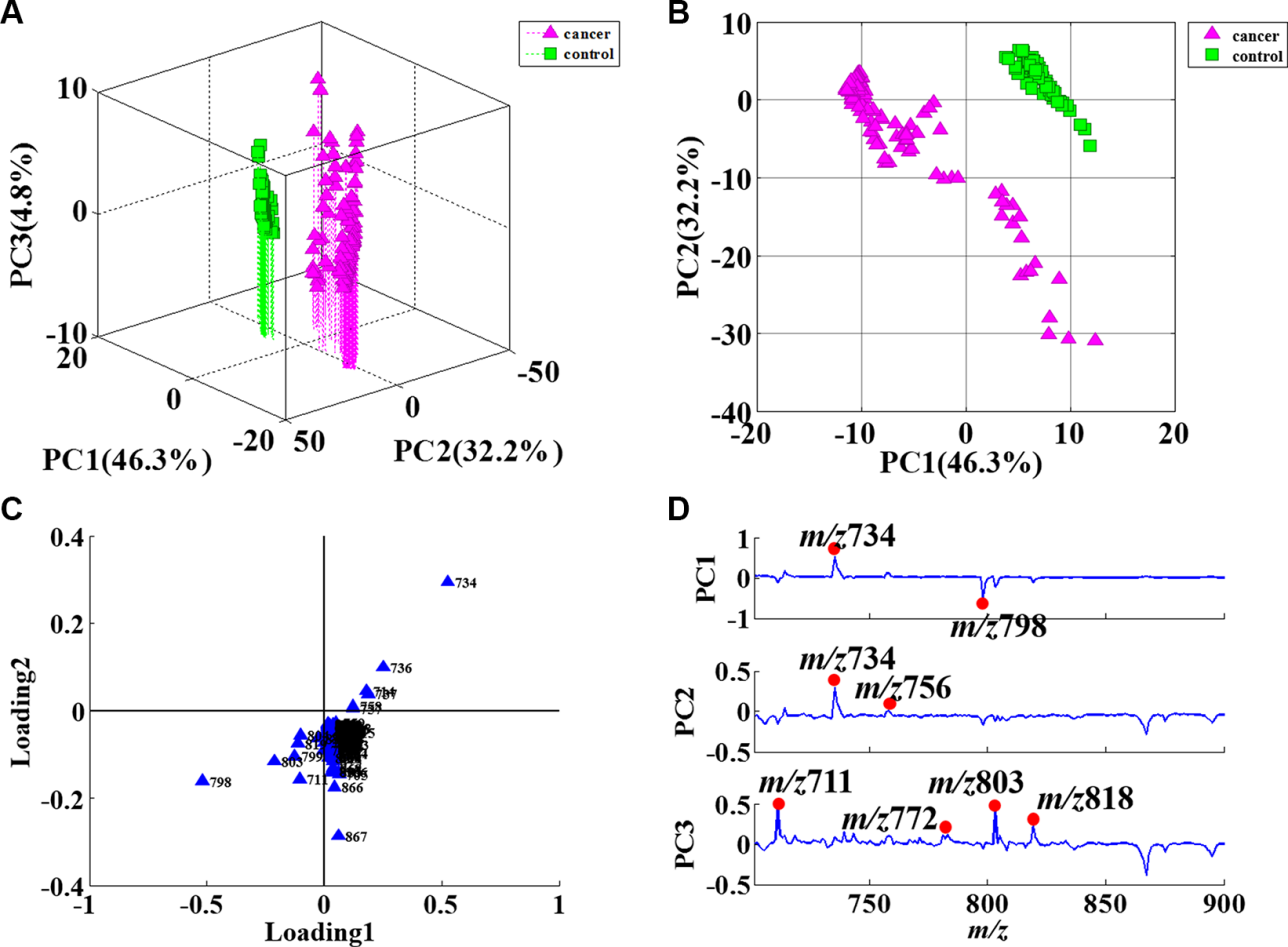
PCA results of sputum in positive ion mode (**A**) 3D plot of PCA score results; (**B**) 2D plot of PCA score results; (**C**) Contribution degree of ions in loading1 and loading2; (**D**) PCA loading results for the PCs. The main differences were in the m/z 711, m/z734, m/z756, m/z772, m/z798, m/z803 and m/z818 ions.

**Figure 3:**
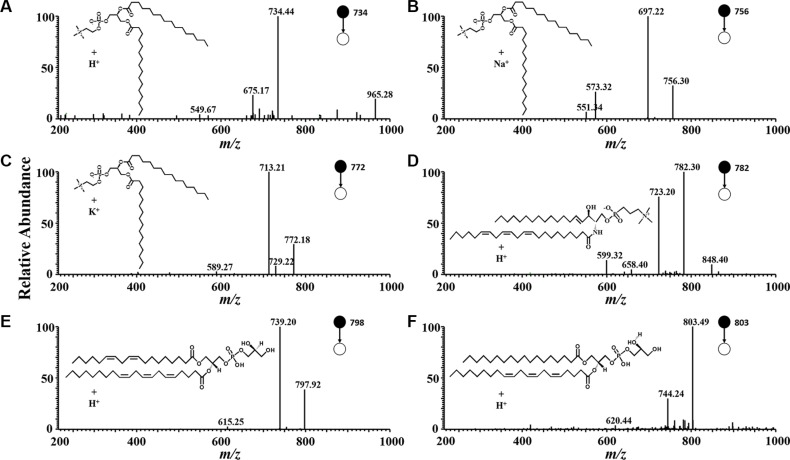
Product ion mass spectra use in CID The product ion mass spectra used in multiple reactions monitoring for: (**A**) dipalmitoyl phosphatidylcholine, [DPPC + H]^+^ (precursor ion m/z 734); (**B**) dipalmitoyl phosphatidylcholine, [DPPC + Na]^+^ (precursor ion m/z 756) ; (**C**) dipalmitoyl phosphatidylcholine, [DPPC + K]^+^ (precursor ion m/z 772); (**D**) sphingomyelin (SM), [SM + H]^+^ (precursor ion m/z 782); (**E**) phosphatidylglycerol (PG), [PG + H]^+^ (precursor ion m/z 798); (**F**) phosphatidylglycerolphosphate (PGP), [PGP+ H]^+^ (precursor ion m/z 803).

PCA is the statistical tool used to explain differentiation between samples and to obtain more information on the variables that mainly influence the sample similarities and differences. This procedure extracts the dominant patterns in the data matrix in terms of a complementary set of scores and loading plots. PCA permits us to achieve a reduction in dimensionality, data exploration for finding relationship between objects, an estimation of the correlation structure of the variables and an investigation of how many components (a linear combination of original features) are necessary to explain the greater part of variance with a minimum loss of information. In this study, PCA was performed on the whole set of average values, with 46.3% in the first principal component (PC1), 32.2% in the second (PC2), and 4.8% in the third (PC3) (Figure 2A, 2B). The three principal components (PCs) explained 83.3% of the total variability. The different ions were mainly the m/z 711, m/z734, m/ z756, m/z772, m/z798, m/z803, and m/ z818, as shown in Figure 2C and 2D. Some ions, including m/ z734, m/z756, m/z772, m/z782, m/z798, and m/z803, were identified and confirmed by CID (Figure 3). The m/z734, m/z756, and m/z772 were [DPPC+H]^+^, [DPPC+Na]^+^, and [DPPC+K]^+^, respectively. And the m/z782, m/z798, and m/ z803 were sphingomyelin (SM), phosphatidylglycerol (PG), and phosphatidylglycerolphosphate (PGP), respectively.

### The change in lipids in the sputum of NSCLC

**Figure 4:**
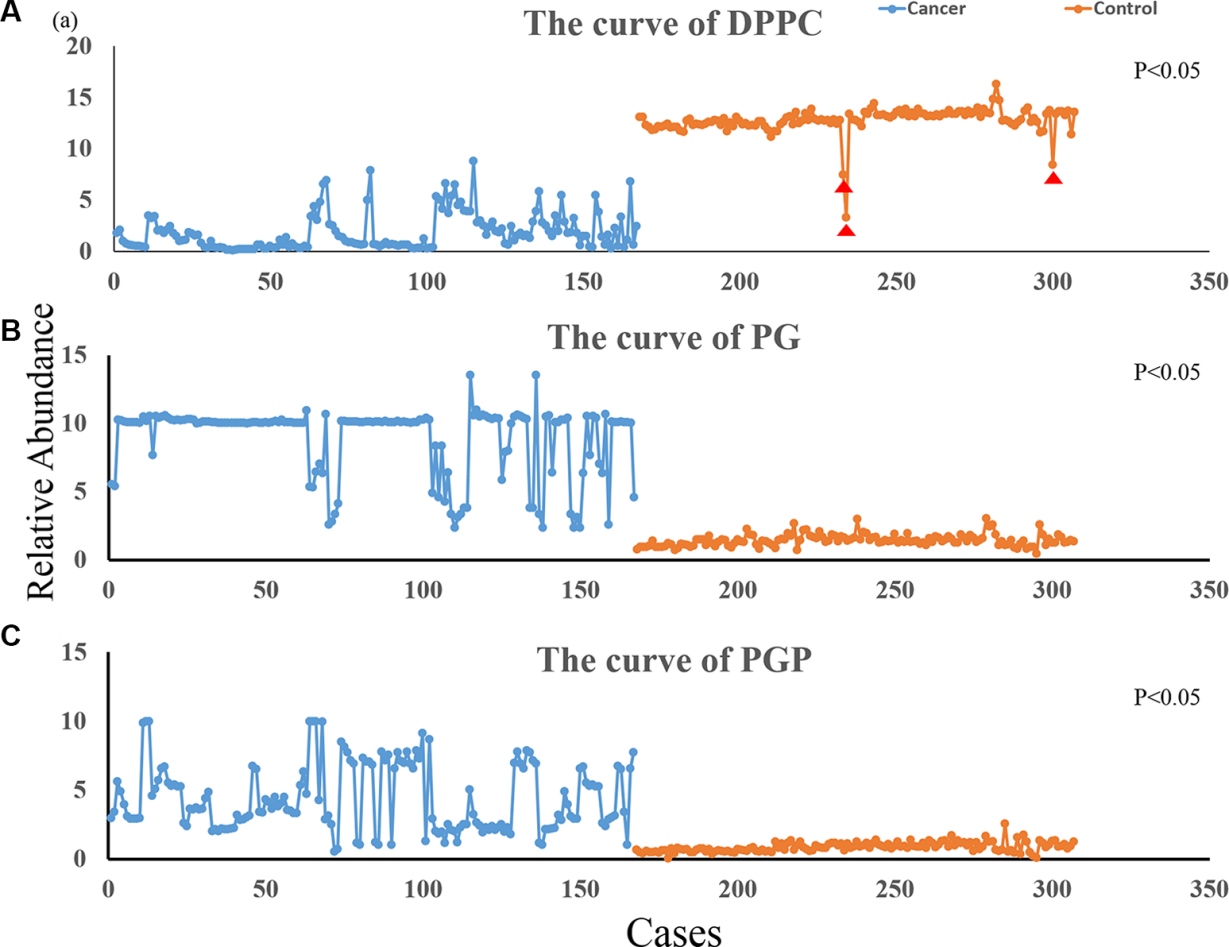
Variation curve for differential ions The blue line represents the relative abundance of ions in NSCLC sputum and the orange line represents the abundance of ions in control sputum; (**A**) is the curve of DPPC (1.83 ± 3.18 vs. 12.78 ± 1.55; *P* < 0.05); (**B**) is the curve of PG (8.92 ± 6.53 vs. 1.42 ± 0.19; *P* < 0.05); (**C**) is the curve of PGP (4.35 ± 6.18 vs. 0.81 ± 0.09; *P* < 0.05); (DPPC were the sum of the m/z734, m/z756 and m/z772 ions, PG was m/z798 ion and PGP was m/z803 ion). Indicates the contols with values that are significantly lower than the average; these may be high-risk cases of lung cancer, and we intend to follow-up these individuals.

According to the results of the PCA, we selected the ions that most significantly contribute to distinguish between NSCLC sputum and control sputum. We imported the relative abundance of the ions into MS Excel, and used Origin 9.0 to chart the curves of the lipids. Figure 4A shows the curves of the DPPC (the sum of the m/z734, m/z756, and m/z772); Figure 4B and 4C show the curves for PG (m/z798) and PGP (m/z803), respectively. The blue line represents NSCLC sputum and the orange line represents the control sputum. The relative abundance of DPPC is clearly lower in NSCLC sputum than in control sputum, though there are three cases among the controls with values that are significantly lower than the average (Figure 4A). In contrast, the relative abundances of PG and PGP are higher in NSCLC sputum than in control sputum (Figure 4B and 4C). The relative abundances of DPPC, PG, and PGP in NSCLC sputum vs. control sputum were 1.83 ± 3.18 vs. 12.78 ± 1.55 (*P* < 0.05), 8.92 ± 6.53 vs. 1.42 ± 0.19 (*P* < 0.05), and 4.35 ± 6.18 vs. 0.81 ± 0.09 (*P* < 0.05), respectively. The relative abundance of SM was no obvious difference between NSCLC and control sputum (0.61± 0.45 vs. 0.74 ± 0.08, *P* > 0.05).

## DISCUSSION

Currently, the main modalities for diagnosis of lung cancer include chest radiography, computerized tomography (CT), bronchoscopy, endoscopic ultrasound (EUS), and endobronchial ultrasound (EBUS). These are not only expensive and invasive but also have low sensitivity for detection of early stage cancer [10].

Sputum is produced in the alveoli and carries much biological information. It is therefore the perfect sample for diagnosis of lung diseases; however, low sensitivity, time-taking procedures, and the need for complicated pretreatment limits its clinical application [5, 11].

MS is being increasingly applied for trace analysis in subcellular metabolism [12], for assessing protein turnover in cell [13], and for disease diagnosis, because of advantages such as rapidity of analysis, high sensitivity, and molecular specificity [6]. Many methods for analysis of sputum by MS have been reported, including two-dimensional gas chromatography time-of-flight MS (GCxGC-TOFMS) [14], gas chromatography–mass spectrometry (GC–MS) [15], and liquid chromatography–tandem mass spectrometry (LC–MS) [16]. However, these methods require complicated pretreatment of samples, such as sputum culture and liquid-liquid extraction; besides, the analysis is time-taking and the operation complicated [3, 16, 17]. Therefore, a method to analyze sputum rapidly and directly is urgently needed. We have earlier reported the use of ND-EESI-MS for direct detection of antibiotics in honey [9]. In view of the similar physical properties of honey and sputum, we decided to apply ND-EESI-MS for the direct analysis of sputum of NSCLC patients.

The identification of molecular markers as a means of early detection of lung cancer has a number of advantages. Unlike the main diagnostic modalities that are currently used, the detection of molecular biomarkers [18] (e.g., proteins, lipids, fatty acids, and amino acids) does not involve invasive procedures or radiation exposure, is relatively inexpensive and repeatable, and may be more efficient for early diagnosis of disease. Indeed, markers such as the DNA methylation status of certain genes can be detected in biofluids such as blood and sputum from people with lung cancer and bronchoalveolar lavage fluid [19–21]. At the molecular level, lipids have an important role in signaling, functioning as second messengers and as hormones [22]. There is increasing evidence that cancer cells show specific alterations in different aspects of lipid metabolism, and changes in lipid metabolism can affect numerous cellular processes, including cell growth, proliferation, differentiation, and motility [23, 24]. In this study, we have detected the differential expression of lipids in NSCLC sputum and control sputum, and propose that this change may be closely related to the onset of NSCLC.

PCA is the statistical tool used to explain differentiation between samples and to obtain more information on the variables that mainly influence sample similarities and differences. This procedure extracts the dominant patterns in the data matrix in terms of a complementary set of scores and loading plots. PCA permits us to achieve a reduction in dimensionality, to explore data for revealing relationships between objects, to estimate the correlation structure of the variables, and to investigate how many components (a linear combination of original features) are necessary to explain the greater part of variance with a minimum loss of information. The PCA results in this study indicated that the ions in NSCLC sputum and control sputum were mostly similar, with only a few unique compounds in NSCLC sputum being suitable for consideration as biomarkers. The total variability was 83.3% in this study, which suggested that the main ions were significantly different between NSCLC sputum and control sputum, and that therefore it was possible to distinguish between the two using ND-EESI-MS and PCA. We found that the m/z734, m/z756, m/z772, m/z798, m/z803, and m/z818 were the main ions that helped distinguish between the two groups in this study. These ions were identified and confirmed by CID. The m/z734, m/z756, and m/z772 were [DPPC + H]^+^, [DPPC + Na]^+^, and [DPPC + K]^+^, respectively. DPPC is a major constituent of pulmonary surfactant. The m/z798 was PG and m/z803 was PGP. PGP and PG are present at levels of 1%–2% in most animal tissues, but it can be the second most abundant phospholipid in lung surfactant, constituting up to 11% of the total (The data comes from Human Metabolome Database: http://www.hmdb.ca/metabolites).

Pulmonary surfactants are produced by type II alveolar epithelial cells (AT II cells) that are a type of lung stem cell. There is evidence that the occurrence of lung cancer is closely related to mutation in these cells [25]. In our previous studies, we have shown that there is relative abundance of DPPC were reduced in cancerous tissue [6]. In the present study, the relative abundance of DPPC was reduced in NSCLC sputum. In addition, the relative abundances of PG and PGP were higher in NSCLC sputum than in control sputum. We are therefore convinced that DPPC, PG, and PGP in sputum are potential molecular markers for lung cancer. Our results demonstrate that ND-EESI-MS can extract the interior chemicals of sputum without the need for any pretreatment [9], and that the differences between two groups of samples can be elucidated with PCA.

In conclusion, we have shown that using ND-EESI-MS in combination with PCA can facilitate the discovery of different lipids, including DPPC, PG, and PGP, in sputum, and that DPPC, PG, and PGP may be useful potential biomarkers of NSCLC. This difference in lipids in sputum can be used to noninvasively predict and assess the occurrence of NSCLC. The method could improve the rate of the early detection of NSCLC and thus improve the survival time of these patients.

## MATERIALS AND METHODS

This study was approved by the Medical Ethics Committee of the Hospital Institutional Review Board of the Second Affiliated Hospital of Nanchang University, Nanchang, People's Republic of China. All clinical investigations were conducted according to the principles expressed in the Declaration of Helsinki.

For this study we used a LTQ-XL mass spectrometer (Thermo Fisher Scientific, San Jose, CA, USA). Methanol (analytical reagent grade) was obtained from Tedia Company Inc., Fairfeild, OH, USA, and acetic acid from Xilong Chemical Company, Shantou, People's Republic of China. The deionized water was homemade. Sputum samples were obtained from the Second Affiliated Hospital of Nanchang University.

**Figure 5:**
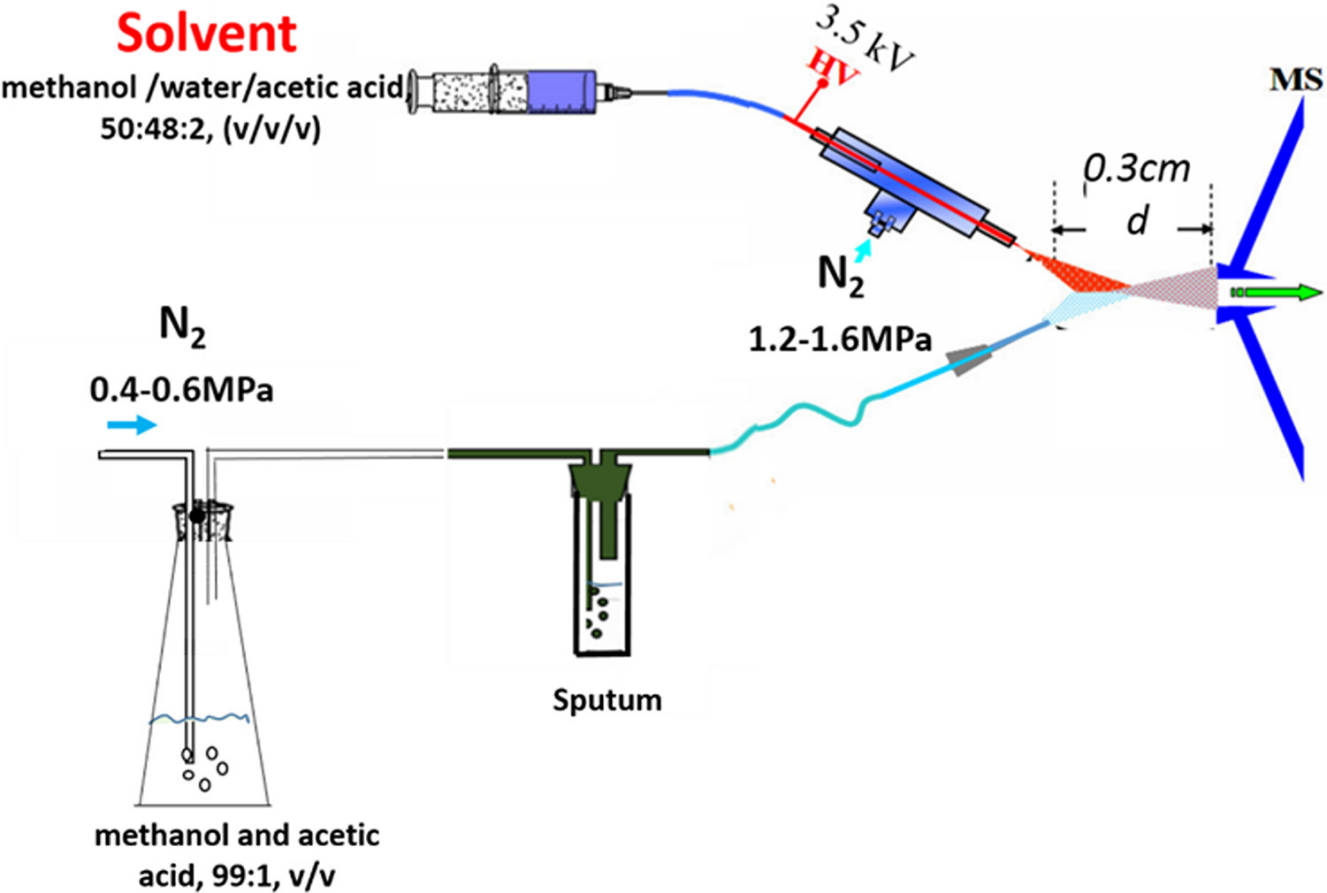
The schematic diagram: A gentle N_2_ gas flow was driven into the desorption liquid to form a N_2_/methanol/acetic acid gas flow as the neutral desorption airflow The analytes were extracted when the neutral desorption airflow was driven into sputum. The ionizing solvent was delivered by a pump, ionized by a high voltage, and atomized by another strong N_2_ gas flow. The interior analytes of the sputum were charged in the ionization region.

The ND apparatus and the EESI ion source were developed by Jiangxi Province Key Laboratory of Mass Science and Instrument. The EESI source (Figure 5) and the LTQ mass spectrometer were set to work in positive ion detection mode. The ionizing solvent (methanol/water/acetic acid, 50:48:2, v/v/v) was delivered at a flow rate 5 μL·min^−1^ by a pump, ionized by a high voltage of +3.5 kV, and atomized by a N_2_ gas flow of 1.2–1.6 MPa. A gentle N_2_ gas flow of 0.4–0.6 MPa was then driven into the desorption liquid (methanol and acetic acid, 99:1, v/v) to form a N_2_/methanol/acetic acid gas flow as the neutral desorption airflow, which was used to bring the interior analytes of the sputum to the ionization region. The interior analytes of sputum were charged continuously when it met the ionizing solvent (Figure 5). The capillary temperature was set to 150°C, the capillary voltage to 35 V, and the tube lens voltage to 100 V. For collision-induced dissociation (CID), the MS/MS spectrum for ion detection used a maximum injection time of 100 ms. The ion selection operation width was 1 Da, and the normalized collision energy was 13%–21%. Other parameters were set as the default values of the instrument, and no further optimization was performed.

### Patients and sample collection

All hospitalized patients were enrolled in this study from March 2015 to May 2016 if they had a diagnosis of NSCLC (confirmed by pathology), no other accompanying malignancies, no other lung diseases, and no history of preoperative chemotherapy or radiotherapy. In total, 167 NSCLC patients were enrolled in this study. The median age of the participants was 59.5 years (range, 38–78 years). There were 87 adenocarcinomas, 75 squamous cell carcinomas, and 5 large cell lung carcinomas in this group. According to the latest lung cancer staging system, 13 patients had stage I disease, 26 had stage Ib disease, 51 had stage IIa disease, 47 had stage IIb disease, 21 had stage IIIa disease, and 9 had stage IIIb disease. The control group (*n* = 140) comprised 43 healthy individuals and 97 patients with other (nonmalignant) lung diseases attending hospital during the same period; the latter included 39 patients with COPD, 33 patients with bronchiectasis, and 25 patients with pneumonia or other bacterial pulmonary diseases. None of these patients had any type of cancer. The median age of the controls was 54 years (range, 34–75 years).

All cases were requested to provide an early morning sputum sample, which was then stored at −80°C until use. Samples were thawed to indoor temperature before ND-EESI-MS analysis.

### Data analysis

Mass spectra were collected in single-stage MS, positive ion mode, and in the mass range of m/z 700–900. Spectral data were binned using a 0.01-T bin size for high-resolution experiments and a 1.0-T bin size for low-resolution experiments, and were stored in a structured query language (SQL) database (Oracle, Redwood City, CA, USA). PCA of the mass spectral fingerprint data was processed using MATLAB, version 7.11 (MathWorks Inc., Natick, MA, USA). PCA transforms the original measured variables into new uncorrelated variables called principal components. The first component describes most of the variation in the data. The second principal component is orthogonal and covers much of the remaining variation. Analyzed data were then standardized to account for the different magnitudes, so that the responses and parameters contributed equally to the data set variance and to the principal component calculation. The ions intensity of differential ions were selected and imported into MS Excel 2013 (Microsoft, Redmond, WA, USA), and the curve was produced in Origin 9.0 (OriginLab Corp., Northampton, MA, USA).

## SUPPLEMENTARY MATERIAL FIGURES


