# Supplementary material for: Altered phosphatidylcholines expression in sputum for diagnosis of non-small cell lung cancer
Source: Oncotarget. 2016 Aug 13;7(39):63158–65. doi: 10.18632/oncotarget.11283 (PMC5325353; doi:10.18632/oncotarget.11283)
Supplement: Supplementary file 1 [file oncotarget-07-63158-s001.pdf]

Altered phosphatidylcholines expression in sputum for diagnosis of non–small cell lung cancer

Supplementary Materials

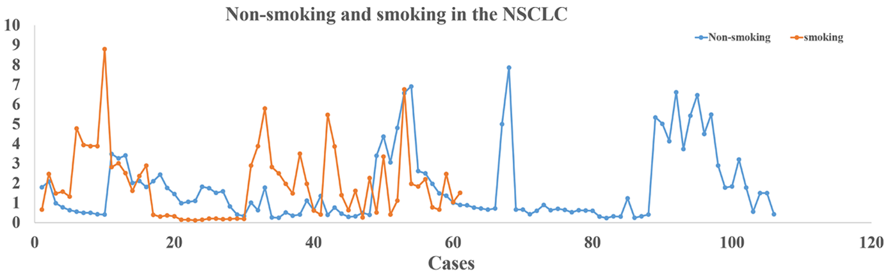

The relative abundances of DPPC (Non-smoking vs smoking: **1.95±3.30 *vs* 1.76±3.14, P>0.05)**

Supplementary Figure S1: The relative abundance of DPPC was no obvious difference between nonsmokers and smokers in the NSCLC group. The relative abundances of DPPC was 1.95 ± 3.30 in nonsmokers vs. 1.76 ± 3.14 in smokers ($P > 0.05$).

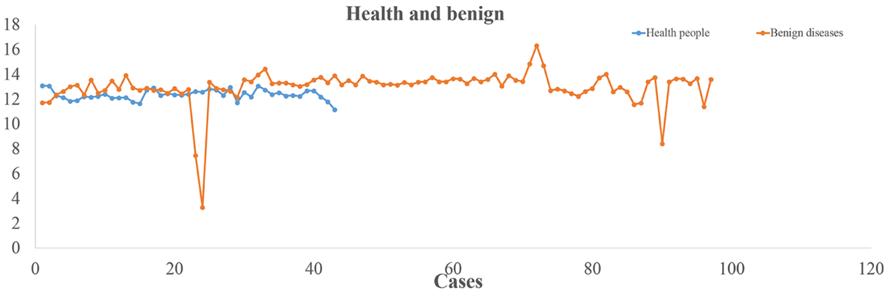

The relative abundances of DPPC (Health vs Benign: **12.35±0.17 *vs* 12.98±2.04, P>0.05)**

Supplementary Figure S2: The relative abundance of DPPC was no obvious difference between sputum of healthy controls and that of patients with nonmalignant lung disease. The relative abundances of DPPC was 12.35 ± 0.17 in healthy controls vs. 12.98 ± 2.04 in patients with nonmalignant lung disease ($P > 0.05$).

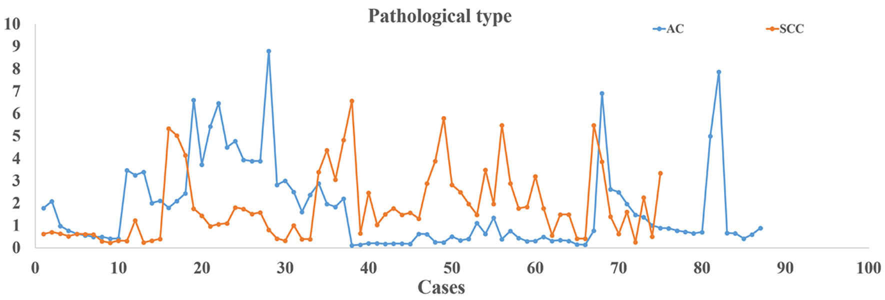

The relative abundances of DPPC (AC *vs* SCC, 1.77±3.65 *vs* 1.87±2.50 *vs* **, P>0.05)**

Supplementary Figure S3: The relative abundance of DPPC was no obvious difference between patients with adenocarcinomas (AC) and those with squamous cell carcinomas (SCC). The relative abundances of DPPC was 1.77 ± 3.65 in patients with adenocarcinomas (AC) vs. 1.87 ± 2.50 in patients with squamous cell carcinomas (SCC) ($P > 0.05$).

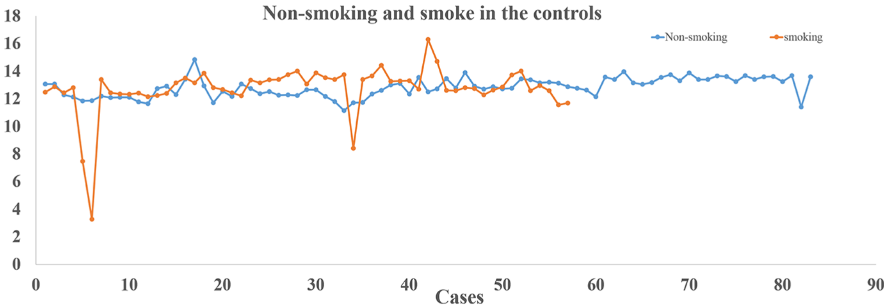

The relative abundances of DPPC (Non-smoking vs smoking: **12.83±0.48 *vs* 12.72±3.13, P>0.05)**

Supplementary Figure S4: The relative abundance of DPPC was no obvious difference between nonsmokers and smokers in the control group. The relative abundances of DPPC was 12.83 ± 0.48 in nonsmokers vs. 12.72 ± 3.13 in smokers ($P > 0.05$).

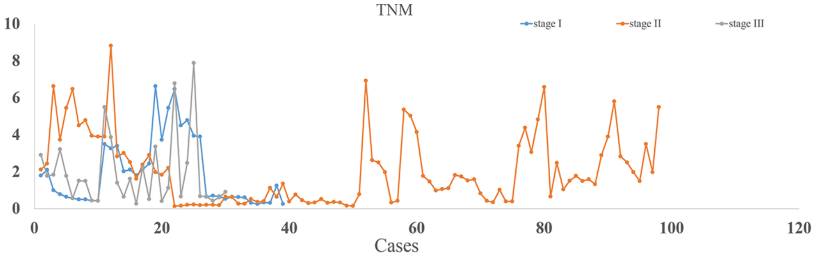

The relative abundances of DPPC (stage I ***vs*** stage II ***vs*** stage III: 1.94±3.36 *vs* 2.07±3.67 *vs* 1.93±3.67, **P>0.05)**

Supplementary Figure S5: The relative abundance of DPPC was no obvious difference in sputum of patients with stage I, stage II, and stage III NSCLC. The relative abundances of DPPC was 1.94 ± 3.36 in stage I , 2.07 ± 3.67 in stage II and 1.93 ± 3.67 in stage III, respectively($P > 0.05$).
